# Diethylstilbestrol Exposure in Neonatal Mice Induces Changes in the Adulthood in the Immune Response to *Taenia crassiceps* without Modifications of Parasite Loads

**DOI:** 10.1155/2014/498681

**Published:** 2014-08-28

**Authors:** Karen E. Nava-Castro, Jorge Morales-Montor, Alejandra Ortega-Hernando, Ignacio Camacho-Arroyo

**Affiliations:** ^1^Facultad de Química, Departamento de Biología, Universidad Nacional Autónoma de México, Ciudad Universitaria, 04510 México, DF, Mexico; ^2^Departamento de Inmunología, Instituto de Investigaciones Biomédicas, Universidad Nacional Autónoma de México, 04510 México, DF, Mexico

## Abstract

Industrial growth has increased the exposition to endocrine disruptor compounds (EDC's), which are exogenous agents with agonist or antagonist action of endogenous steroid hormones that may affect the course of parasite infections. We wanted to determine if the exposure to diethylstilbestrol (DES), an estrogen agonist, to both male and female mice affected the immune response and their susceptibility to *T. crassiceps* cysticercosis. In all infected groups, females showed higher parasite loads than males, and neonatal DES administration did not modify this pattern. In the spleen, noninfected mice showed sex-related differences in the percentage of the CD8+ subpopulation, but DES decreased the percentage of CD3+, CD19+, and CD8+ subpopulations in infected mice. In the mesenteric lymphatic node (MNL), DES showed a dimorphic effect in the percentage of CD19+ cells. Regarding estrogen receptor alpha (ER-α) expression, DES treatment induced a reduction in the expression of this receptor in both noninfected female and male mice in the spleen, which was decreased only in males in CD3+ and CD8+ lymphocytes in MNL cell subpopulations. Our study is the first one to demonstrate that DES neonatal treatment in male and female mice affects the immune cell percentage, without effect on the susceptibility to *T. crassiceps* cysticercosis.

## 1. Introduction

Endocrine disruptor compounds (EDC's) are exogenous agents that interfere with the synthesis, secretion, transport, binding, action, or elimination of natural hormones in the body with agonist or antagonist action of endogenous hormones. EDCs are from natural sources such as xenoestrogens or have a chemical origin such as diethylstilbestrol (DES), Bisphenol A (BPA), TCDD, and DTT among others [1]. In particular, DES was administered to millions of pregnant women to prevent miscarriages caused by progesterone deficiency between 1940 and 1971 [2]. Studies on neonatal treatment with DES in animal models have reported negative effects on the normal morphology and physiology of the reproductive tract [3, 4]. Several studies have also demonstrated that DES exposure during the fetal and prenatal stages induces tumor formation on estrogen-sensitive tissue in several mice and hamsters models. In adult mice, DES administration also induces cancer in mammary gland, cervix, and uterus. It can also increase the incidence of leukemia and lymphoid tissue tumors [2, 5].

The effect of EDC's on the immune cell function has been barely studied. In humans, prenatal exposure to some EDC's such as DES increased lymphocyte proliferation in response to some chemical mitogens such as Concanavalin A or phytohemagglutinin [5]. DES administration at gestational eighteen day in mice also reduces thymocyte number without changes in thymocyte subpopulations [6].

In experimental murine cysticercosis caused by* Taenia crassiceps* [7, 8], females of all strains of mice studied sustain larger intensities of infection than males [9]. 17*β*-estradiol (E_2_) promotes cysticercus growth by interfering with the thymus dependent cellular immune mechanisms [10]. In addition, gonadectomy alters the resistance pattern of males and produces similar intensities of infection in both sexes [11]. When gonadectomized males receive hormonal replacement with testosterone (T_4_), parasite loads are reduced, while E_2_ replacement in female mice increased parasite loads [10].

The immune cells mature during fetal development and are exposed to their first external antigens during the neonatal stage. Thus, exposure to EDCs at this early stage of development may affect effectors of the immune response. We have previously demonstrated that neonatal exposure (postnatal day 4) to E_2_ increases the resistance to* T. crassiceps* infection in both male and female mice during adulthood. This resistance was accompanied by an increase in the expression of IL-4 and IFN-*γ* in the serum of experimentally infected neonatally estrogenized animals [12].

At present, however, it is not known whether the administration of DES during the critical period of sexual differentiation of the brain affects the activity of the immune system.

Experimental murine* T. crassiceps* cysticercosis has contributed to revealing the complexities of the interactive network that regulates infection, which is formed by the immune and neuroendocrine systems of the host and the parasite [13]. Briefly, remarkable sex-associated susceptibility to* T. crassiceps *cysticercosis occurs in mice, with females developing larger parasite loads than males during early infection [14]. After 4 weeks of infection, the parasite loads in males slowly increase and within a few months they approximate the parasite loads found in females [15]. Concomitantly, a feminization process occurs in chronically infected male mice, in which serum E_2_ levels progressively increase until they almost reach those observed in females. After 6 weeks of infection, T_4_ levels drop to 10–15% of normal male levels [15–17] and infected male mice progressively lose their normal aggressive mating behavior [16, 18]. High E_2_ levels correlate with an increase in* c-fos* transcription factor and mRNA expression in different areas of the brain at different times of infection [13].

Because sex hormones play a fundamental role in the development of the* T. crassiceps *infection, we hypothesized that a change in the hormonal microenvironment of the host induced by a neonatal injection of DES could determine the proportion of immune cell subpopulations and its estrogen receptors expression during adulthood. This should result in a change in susceptibility to infection in mice, through the modification of immunity of the host towards the parasite. Thus, the aim of this study was to investigate changes in the proportion of immune cells in spleen, mesenteric nodes, and expression of estrogen receptors after a single neonatal dose of DES during* T. crassiceps *infection and to correlate these data with the parasite burden in male and female Balb/c mice. Our findings support the key role of neonatal exposure to DES in controlling immune system function, but not susceptibility to infection in mice.

## 2. Materials and Methods

### 2.1. Ethics Statement

The Animal Care and Use Committee at the Instituto de Investigaciones Biomédicas evaluated animal care and experimentation practices according to the official Mexican regulations (NOM-062-ZOO-1999) in strict accordance with the recommendations in the Guide for the Care and Use of Laboratory Animals of the National Institutes of Health (NIH) of the USA. The Ethics Committee of the Instituto de Investigaciones Biomédicas approved this protocol (Permission Number 2009–13).

### 2.2. Animals

Male and female Balb/cAnN (H2-d) inbred mice obtained from Harlan (Mexico City) were used in all experiments. Animals were housed in the animal care facilities at Instituto de Investigaciones Biomédicas, (UNAM), under controlled conditions of temperature and 12 h dark-light cycles with lights on between 0700 and 1900.

### 2.3. Neonatal Injection of DES

One single dose of DES was subcutaneously injected (5 *μ*g per mouse) into 4-day-old mice of both sexes. The dose and time of infection were based on previous report by our group [12]. Vehicle—mineral oil—was similarly administered to another group of mice. Intact age matched mice were kept as untreated controls.

### 2.4. Experimental Infections

At 6 weeks of age, ten nonbudding* T*.* crassiceps* larvae of the fast-growing ORF strain [19] (approximately 2 mm in diameter) were suspended in 0.3 mL sterile phosphate-buffered saline (PBS: 0.15 M NaCl, 0.01 M sodium phosphate buffer, pH 7.2) and intraperitoneally injected into each male and female mouse using a 0.25 gauge needle. Noninfected mice of each sex were used as age-matched controls. Mice were rapidly euthanized by sevoflurane inhalation (Abbott, Mexico) at 8 weeks of infection. Peritoneal cysticerci were collected and counted after rinsing the peritoneal cavity with PBS. Spleen and mesenteric lymphatic nodes were collected immediately after rinsing, to use in flow cytometry assays.

### 2.5. Flow Cytometry

Briefly, splenocytes from BALB/c mice were purified and stained with the following antibodies: anti-mCD3-FITC, mCD3-biotin, mCD4-APC-Cy7, mCD8-PECy5, and mCD19-PE (from Biolegend). Streptavidin-APC was used as a secondary reagent for CD3-biotin. Cells were fixed with 500 *μ*L of Fixation buffer (4% Paraformaldehyde, Ix PBS, pH. 7.4) and then permeabilized with 200 *μ*L of Perm Buffer (0.2% Saponin, 4% FBS, 1 mM NaN_2_). After washing, cells were incubated with Fc blocking reagent (CD16/CD32-FcgammaIII/II Receptor) and incubated with purified anti-ER-α (estrogen receptor alpha) (Santa Cruz Biotech) in Perm Buffer. Primary antibodies were detected with Alexa488- or Alexa647-coupled secondary antibodies (Biolegend) incubated in Perm Buffer. Cells were finally washed with FACS buffer (2% FBS, 0.02% NaN_2_, Ix PBS, pH. 7.4) and stored until analyzed. Samples were analyzed by flow cytometry using a FACSAria (BD Biosciences) and data were analyzed with the FlowJo software. Relative ER expression was calculated as follows: media fluorescence intensity (MFI) MFI = MFI from ER-stained samples/MFI from the secondary antibody-stained sample from the same tissue and mouse. Mean values ± SEM are shown.

### 2.6. Experimental Design and Statistical Analysis

The experimental design was a four factorial experiment. Independent variables were (1) neonatal DES injection (two levels: yes or no); (2) tissue under study (two levels: spleen, mesenteric lymph nodes); (3) infection (two levels: yes, no); (4) sex (two levels: male or female). The dependent variables were percentage of immune subpopulations, ER expression on each subpopulation, and the number of parasites. Statistical analysis of 2-way ANOVA and Bonferroni's test were performed with the software GraphPad Prism (version 5.0 b for MacOSX, GraphPad Software, San Diego California USA, (http://www.graphpad.com/)).

## 3. Results

### 3.1. Female Puberty Onset and Cyclicity

Vaginal opening was not statistically significant, in neonatally DES treated than in vehicle-treated female mice (29.3 ± 0.3 and 30.3 ± 0.4 days, resp.). Immediately after vaginal opening, neonatally DES treated females showed longer estrous cycles particularly arrested at estrous. After 8 weeks of infection, females did not show estrous cycle regardless of neonatal treatment, while noninfected, age-matched, neonatally DES females continued to exhibit longer cycles compared to vehicle-treated mice (not shown).

### 3.2. Parasite Loads

**Figure 1:**
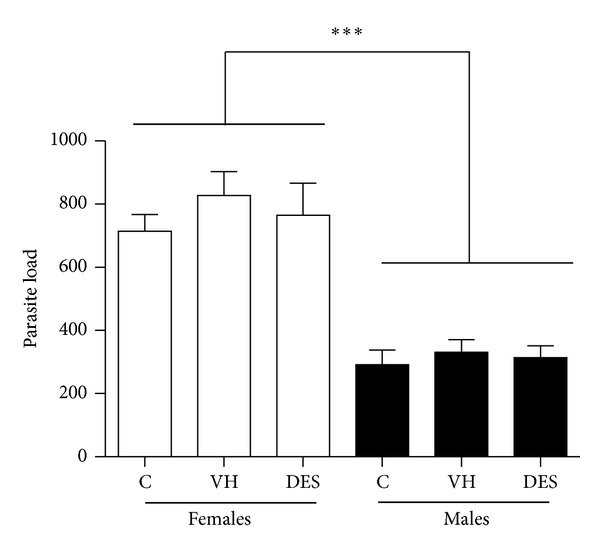
Effect of neonatal administration of diethylstilbestrol (DES) on parasite load. Female and male 4-day old Balb/c mice were administered with a single dose of DES or vehicle (VH) and infected with 10 cysticerci of* Taenia crassiceps *at 6 weeks of age. Data show the number of parasites recovered from the peritoneum at 8 weeks after infection. All groups of animals show the typical sexual dimorphism of this infection. Each bar represents the media ± SEM of parasite loads in 10 infected animals. ∗∗∗*P* < 0.001.

As others and we have previously reported, we confirm and extend the gender related differences in parasite loads (sexual dimorphism) among male and female mice. In all infected groups, females showed higher parasite loads than males. We did not find differences, either in males or females, produced by neonatal DES administration (Figure 1).

### 3.3. Spleen Cell Subpopulations

**Figure 2:**
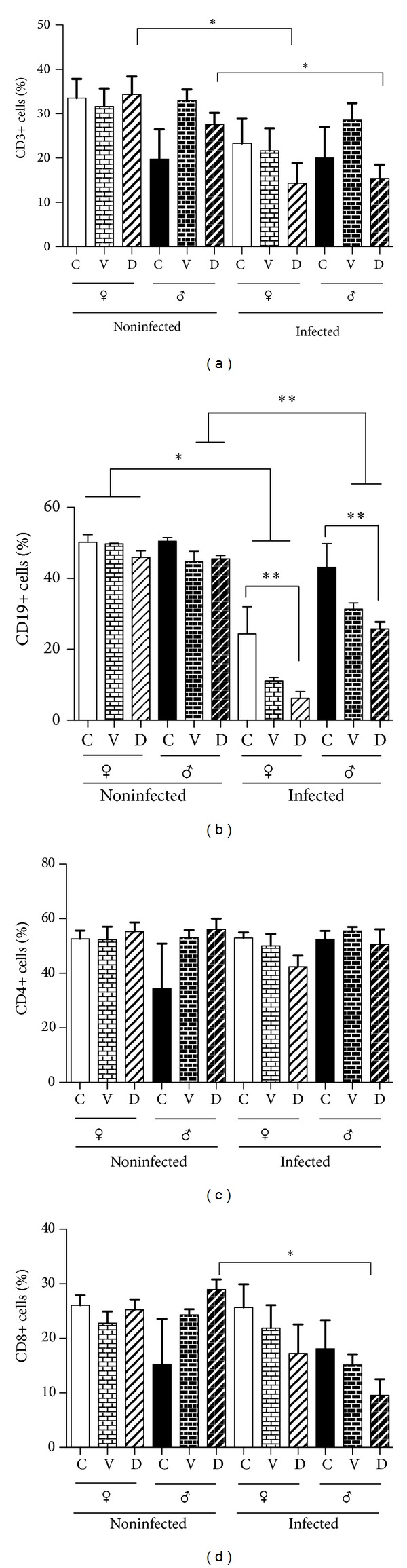
Percentage of lymphocyte populations in spleen of intact (C), vehicle (V), and DES-treated (D) mice, detected by flow cytometry. T lymphocytes (CD3+, CD4+, and CD8+) and B lymphocytes (CD19) were detected in spleen. Each bar represents the media ± SD of parasite loads in 10 infected animals.* Post hoc* individual contrasts of group means by Bonferroni's Exact Test used the sum of residual and three-factor interaction variance to test for significant differences. ∗∗∗*P* < 0.001; ∗∗*P* < 0.01.

In order to study the effects of DES on lymphocyte subpopulations in the spleen, we decided to determine the proportion of each cell type present in this organ and to analyze if their pattern was sexually dimorphic and if it was affected by DES treatment or infection. As shown in Figure 2, noninfected mice showed no differences in the percentage of any of the analyzed subpopulations when comparing between sexes. Regarding DES treatment, it decreased the percentage of CD3+, CD19+, and CD8+ subpopulations in infected mice when comparing to their noninfected counterparts (Figures 2(a), 2(b), and 2(d)). It is interesting to note that DES effect was more pronounced in the CD19+ cell subpopulation and it was higher in females than in males (when comparing control mice versus DES-treated mice) (Figure 2(b)).

### 3.4. Mesenteric Lymphatic Node Cell Subpopulations

**Figure 3:**
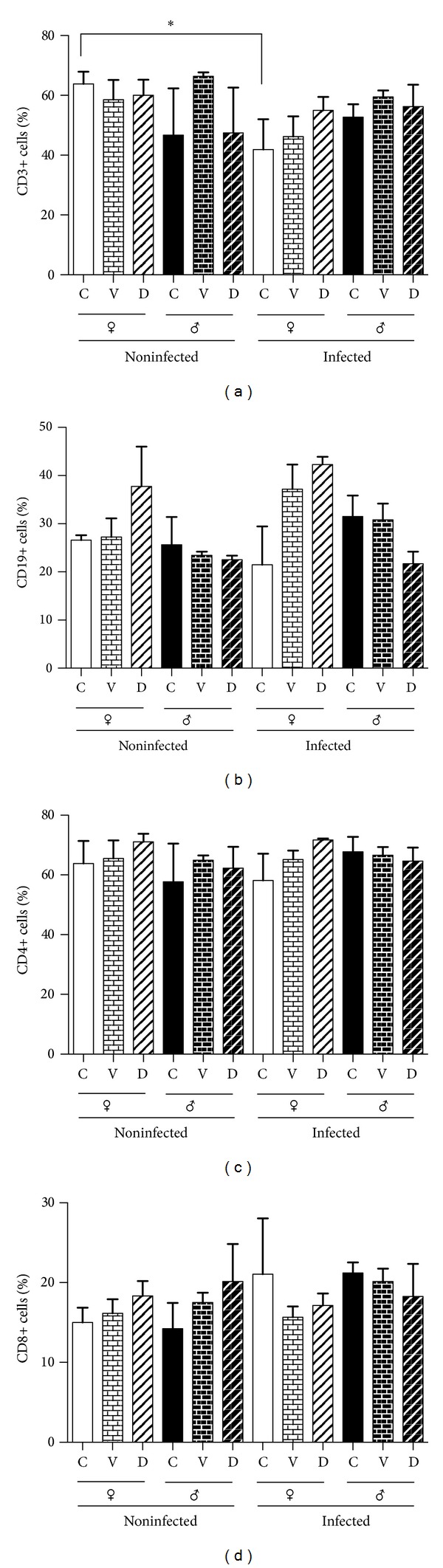
Percentage of immune cell populations in mesenteric lymphatic nodes of intact, vehicle, and DES treated mice, detected by flow cytometry. T lymphocytes (CD3+, CD4+, and CD8+) and B lymphocytes (CD19) were detected in mesenteric lymphatic nodes. Each bar represents the media ± SEM of parasite loads in 10 infected animals.* Post hoc* individual contrasts of group means by Bonferroni's Exact Test used the sum of residual and three-factor interaction variance to test for significant differences. ∗*P* < 0.05.

In order to characterize the subpopulations of lymphocytes present in the mesenteric lymphatic nodes, we decided to determine the percent of each lymphocyte type present in this immune compartment and to analyze if their pattern was dimorphic, affected by DES treatment or the infection. We found no differences in the percentage of the subpopulations in females and males, either in control or infected mice, except for the CD3+ subpopulation in females, where infection induced a significant decrease in this subpopulation (Figure 3(a)). DES treatment showed a dimorphic effect in the CD19+ cells. It induced an increase in infected females while it decreases this subpopulation in infected male mice. However, this difference was not significant. Our FACS analysis showed that infection did not affect the pattern of different subpopulations (Figure 3).

### 3.5. Expression of Estrogen Receptor Alpha by FACS in Each Spleen Cell Subpopulation

**Figure 4:**
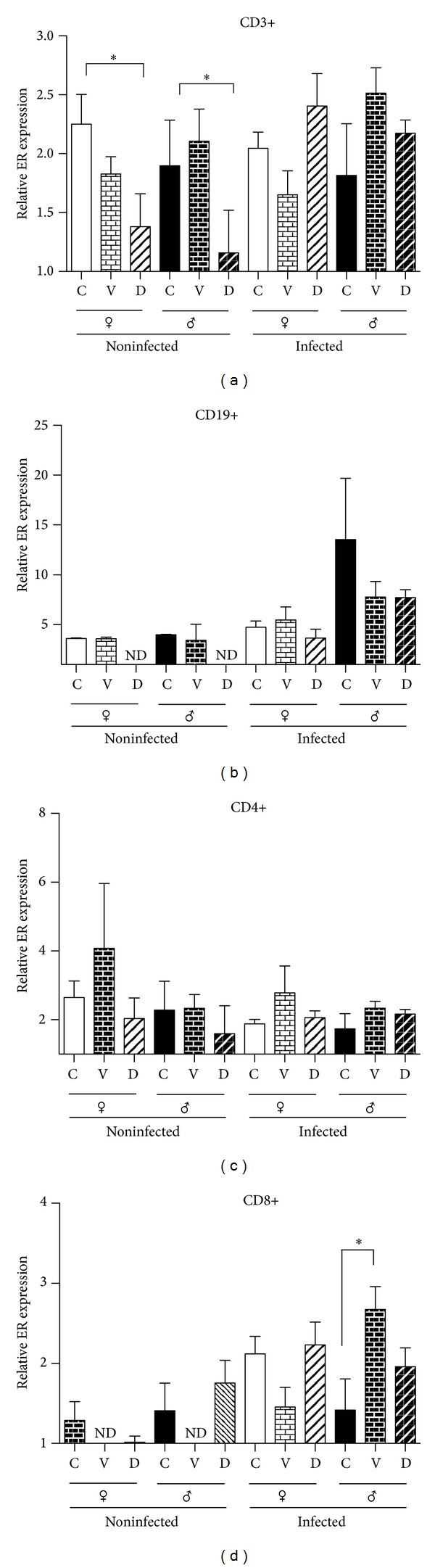
Effects of infection and neonatal DES administration on estrogen receptor α content in splenocytes of mice of both sexes, control, and infected with cysticerci of* Taenia crassiceps *detected by flow cytometry. Estrogen receptor alpha was detected in T lymphocytes (CD3+, CD4+, and CD8+) and B lymphocytes (CD19+). Data are presented as the mean ± SEM of 2 different experiments, *n* = 10 each.

We observed that mice spleen expresses ER-α. We found no differences in the expression of this receptor in any of the analyzed subpopulations in noninfected mice. When the expression of this receptor was measured in DES treated animals, we found that DES induces a reduction in the expression of this receptor in CD3+ and CD19+ cells of both noninfected female and male mice. This reduction is maintained in infected mice when analyzing the CD19+ subpopulation, but in this case, we found no significant differences (Figure 4(b)). We found no differences induced by DES treatment in CD4+ or CD8+ subpopulations neither because of the sex nor the infection (Figures 4(c) and 4(d)).

### 3.6. Expression of Estrogen Receptor Alpha by FACS in Mesenteric Lymphatic Node Cell Subpopulations

**Figure 5:**
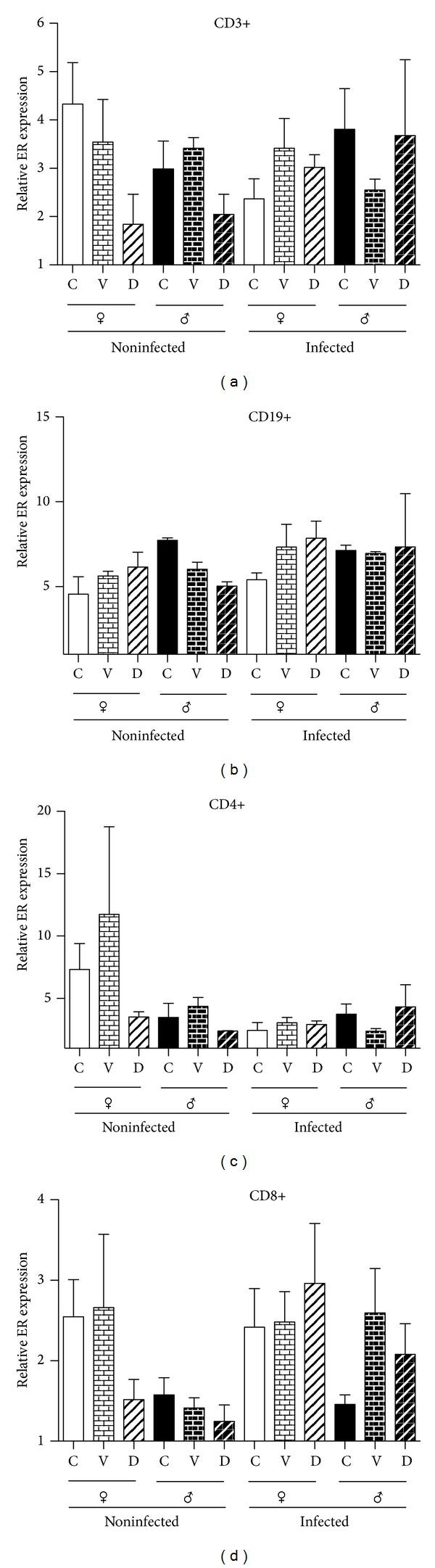
Effects of infection and neonatal DES administration on estrogen receptor α content in mesenteric nodes of mice, control, and infected with cysticerci of* Taenia crassiceps *detected by flow cytometry. Estrogen receptor alpha was detected in T lymphocytes (CD3+, CD4+, and CD8+) and B lymphocytes (CD19+). Data are presented as the mean ± SEM of 2 different experiments, *n* = 10 each.* Post hoc* individual contrasts of group means by Bonferroni's Exact Test used the sum of residual and three-factor interaction variance to test for significant differences.

Lymphocytes of mesenteric lymphatic node cell subpopulations also express ER-α. We also observed that this receptor is dimorphically expressed, since males show a higher expression than females in noninfected mice and in the T cell subpopulations analyzed. This effect was not observed in the B cell subpopulation. When the expression of these receptors was measured in DES treated animals, we found decreased expression of ER just in males in the total lymphocyte subpopulation (CD3+) and cytotoxic lymphocytes (CD8+). However, these differences did not reach significance. Infection did not affect the expression of the ER-α protein in any sex or DES treated animals (Figure 5).

## 4. Discussion

In this study, DES administration to neonatal male and female mice was found to produce few changes in immune cell percentage levels, as well as estrogen receptor expression on different immune compartments without affecting the sex-associated susceptibility to* Taenia crassiceps* infection when mice reached the adult phase. Although many reports on neonatal administration of compounds with estrogenic activity have described negative long-term effects on reproductive function [20–24], the impact of neonatal DES treatment on adult mouse susceptibility to helminth parasites had not been studied so far. Since E_2_ is an endogenous steroid produced by the ovaries, it is important to study the effects that exposure to analogue compounds, such as DES, on immune functions have. Along the same line, pollutants with estrogenic activity that are released into the environment as a consequence of manufacturing processes could interfere with the normal development of regular immune functions during adult life. The developmental windows during which they can cause harmful effects are critical. We have previously found that a single-dose of E_2_ permanently modified immune functions and immune response to* T. crassiceps*. Exogenous steroid administration showed to upregulate the immune system, specifically the cellular immune response, by increasing IL-4 and IFN-*γ* serum protein levels in a sexually dimorphic manner as a response to neonatal E_2_ treatment [12]. In the present study, we confirm and extend the notion that neonatally administered EDCs affect the immune function.

Some studies suggest that E_2_ potentiates the production of cytokines Th1 (IFN-*γ*) and Th2 (IL-10). High E_2_ concentrations were found to stimulate IL-10 secretion by T cell clones, while low concentrations stimulate IFN-*γ* secretion [24, 25]. In women with a regular menstrual cycle, the immune response tends towards a Th2-type response, which is reflected as an increment in IL-4 production [26]. This suggests that the increased E_2_ concentrations during the luteal phase play a role in the deviation of the immune response towards a Th2-type response. This steroid hormone pattern of action was also observed in the lymphocyte proliferation experiments reported by de León Nava et al., 2009 [25]. Lymph node cells were obtained from mice of the two sexes then cultured, activated with anti-CD3 and anti-CD-28, and treated with E_2_, P_4_, and T_4_. E_2_ inhibited lymphocyte proliferation. Female cells proliferated more than male cells; however, paradoxically, precisely the steroids associated to female physiology were those to inhibit lymphocyte proliferation. In the specific case of DES, administration into adult rats increases susceptibility to* T. spiralis* infection, when administrated for 5 days (total dosage: 40 mg/kg) before larvae administration [27]. A number of reports on immune and neuroendocrine system interactions indicate that hormones are capable of affecting immune functions [28]. The importance of the interaction between the immune and endocrine systems becomes evident in circumstances such as pregnancy, autoimmune diseases, and some infectious diseases and, as presented in this report, the exposure to endocrine disruptor. In all cases, available evidence underscores the importance of sex steroids as immunoregulators.

The possible mechanisms of action of steroids on immune system cells include, as in any classic endocrine tissue, the genomic and nongenomic pathways. According to the genomic action theory, steroids bind to specific receptors present in the cytoplasm and function as transcription factors. Besides their genomic action, steroids can also act by rapid nongenomic pathways, and the transmission of these effects occurs by specific membrane receptors. Thus, the nongenomic effects on cell function implicate the conventional cascades of second messengers [29]. Although these mechanisms of action have been described in endocrine system organs, evidence has accumulated that they can also operate in the immune system.

Considering the effects that steroids exert on the diverse components of the immune system and that no previous reports are available on the different receptors present in a peripheral organ such as the spleen, we herein aimed to detect the expression of these receptors in the spleen and lymphatic node lymphocyte subpopulations of control, vehicle, DES-treated, control, or infected of both sexes. Ample distribution of ER-α has been found in spleen [30]; however, published reports on the presence of estrogen receptors in spleen contrast with the few publications on estrogen receptors lymphoid mesenteric nodes tissue. In the present work we found the expression of ER-α in the spleen and lymphoid mesenteric nodes of mice of the two sexes neonatally treated with DES and infected in the adulthood. DES treatment, in turn, had significant sex-associated effect on the expression of this receptor. Although the expression level in spleen is much lower than in endocrine tissue, the presence of this ligand-dependent transcription factor is relevant in a secondary lymphatic organ, since it draws attention to the fact that sex steroids may act not only during the maturation and development of immune cells (in thymus) but also during the effector mechanisms of these cells.

Because the molecular mechanisms by which DES affect the immune system function could be due to their interaction with a specific nuclear receptor, we studied ER-α expression in spleen cells and mesenteric nodes cells of all treatments.

Considering the effects that DES and sex steroids exert on the diverse components of the immune system and that no previous reports are available on the differential expression of this receptor in immune cells in a peripheral organ such as the spleen or mesenteric nodes, as well as its regulation by DES or* T. crassiceps* infection, we herein aimed to detect the expression of this receptor in the spleen and mesenteric nodes of control, vehicle, DES-treated in noninfected and infected mice of both sexes. Ample distribution of ER-α has been found in thymus, bone marrow, and spleen [31–33]; however, published reports on the presence of ER-α in splenic lymphoid tissue contrast with the few publications on its presence on mesenteric lymphatic nodes. In the present work we found the expression of ER-α in the spleen and mesenteric lymphatic nodes of mice of the two sexes. Although the expression level in spleen and mesenteric lymphatic nodes is much lower than in endocrine tissue, the presence of this ligand-dependent transcription factor is relevant in both immune organs, since it draws attention to the fact that DES may act not only during the maturation and development of immune cells but also during the effector mechanisms of these cells. The question about the population of immune cells that expresses receptors and their regulation was also addressed by flow cytometry analysis.

The possible mechanisms of action of DES on immune system cells, being an agonist or antagonist of estrogens (sex steroids), may include, as in any classic endocrine tissue, the genomic and nongenomic pathways. According to the genomic action theory, steroids or molecules similar to steroids, such as DES, bind to specific receptors present in the cytoplasm and function as transcription factors. Besides their genomic action, steroids and agonists or antagonists, such as DES, can also act by rapid nongenomic pathways, and the transmission of these effects occurs by specific membrane receptors. Thus, the nongenomic effects on cell function implicate the conventional cascades of second messengers [32]. Although these mechanisms of action have been described in endocrine system organs, evidence has been accumulated that they can also operate in the immune system. According to the work by Benten et al. [34], the effects of T4 on T cells are mediated not only by the intracellular androgen receptor, but also by a membrane androgen receptor on the cell surface. Thus, DES and steroid hormones may act through intracellular and membrane receptors of immune system cells by the nongenomic pathway, whenever the regulation of an immune response against a particular pathogen requires their immediate action and by the genomic pathway, when the response needs to be delayed. Speculation ensues on the possibility that the nongenomic pathway predominantly regulates the innate immune response, while the genomic pathway does the same with the adaptive immune response.

In addition to the question of which cell population responds to DES, it would also be interesting to determine the age at which the immune system acquires its dimorphic character [25, 35].

The evidence presented above illustrates the importance of immunoendocrine interactions in an immunocompetent host during* Taenia crassiceps* infection. Interventions aimed at the hormonal network appear as a possible new therapeutic approach to control several immune confrontations, such as parasitic infections.
